# Calibrating Trust, Reliance and Dependence in Variable-Reliability Automation

**DOI:** 10.1177/10711813241277531

**Published:** 2024-09-02

**Authors:** Christopher Holland, Grace Perry, Heather F. Neyedli

**Affiliations:** 1 Dalhousie University, Halifax, NS, Canada

**Keywords:** human–automation interaction, decision aid, trust in automation, automation reliability, trust calibration, trust dynamics

## Abstract

Trust and system reliability can influence a user’s dependence on automated systems. This study aimed to investigate how increases and decreases in automation reliability affect users’ trust in these systems and how these changes in trust are associated with users’ dependence on the system. Participants completed a color identification task with the help of an automated aid, where the reliability of this aid either increased from 50% to 100% or decreased from 100% to 50% as the task progressed, depending on which group the participants were assigned to. Participants’ trust, self-confidence, and dependence on the system were measured throughout the experiment. There were no differences in trust between the two groups throughout the experiment; however, participants’ dependence behavior did follow system reliability. These findings highlight that trust is not always correlated with system reliability, and that although trust can often influence dependence, it does not always determine it.

## Introduction

Automated systems are integral to various industries, including military operations, medical diagnosis (Du et al., 2020), aviation, motor vehicle operations, and information retrieval (Lee & See, 2004). Despite their benefits, multiple factors can impact their use, affecting the overall effectiveness and adoption of automated technologies.

The interplay between trust, self-confidence, and system reliability significantly influences users’ dependence on automated systems. Trust in a system is a key predictor of dependence, but it does not solely determine it (Lee & See, 2004). Traditional views of self-confidence have proposed that there are trade-offs between self-confidence, reliability of the system and trust. When users’ trust in the system surpasses their self-confidence in performing the task, they tend to depend more on the automation (Hutchinson et al., 2022; Parasuraman & Riley, 1997). Conversely, if users have higher self-confidence, they are more likely to overlook the system’s assistance (Desai et al., 2012). More recent research using more dynamic measures of trust and self-confidence puts this traditional view into question, showing that individuals with greater self-confidence may display better calibration of their trust to the automation’s capabilities (Williams et al., 2023).

Understanding trust dynamics within the context of automated systems involves examining how users’ trust evolves over time and its impact on their dependence on the automated system given that trust can rapidly change with a single interaction (Chung & Yang, 2024; Yang et al., 2023). Trust calibration is important because inappropriate levels of trust can lead to either over-reliance or underutilization of system capabilities (Merritt et al., 2015). Rittenberg et al. (2024) showed that the direction of continuous changes in system reliability interact with user trust and self-confidence. Separate groups of participants interacted with automation that either increased or decreased between 50% and 100% reliable over the course of 300 trials. Contrary to expectations, trust in the automation decreased in both groups. Similar to Williams et al. (2023), Rittenberg and colleagues also showed that participants with higher self-confidence showed greater change in their trust levels with the changing reliability level. Interestingly, in both groups, participants’ performance tracked reliability level with participants in the increasing group performing better over the course of the 300 trials and vice versa for the decreasing group. This seems to indicate that although automation trust was declining in both groups, participants were still depending on the automation. One limitation however is that performance was used as a proxy for dependence on the automation and dependence was not measured directly.

This study builds on Rittenberg et al. (2024) by studying how increasing or decreasing automation reliability affects dependence. A user’s behavior in utilizing automated systems has often been defined by various terms such as dependence, reliance, and compliance. While all these terms are commonly used to characterize system usage, reliance and compliance generally refer to contexts involving alarm states (Boubin et al., 2017; Chancey et al., 2017; Du et al., 2019; Meyer, 2004; Meyer et al., 2014). Dependence takes a broader approach, referring to users relying on the system overall (Chancey et al., 2017), such as allowing the system to fully influence their decision-making process. Due to the varying definitions and measurements among these terms, this study uses “dependence’ to denote participants” use of the information provided by the system to update and change a user’s prior decision. Dependence and trust are often thought to be related (e.g., Hussein et al., 2020), with increases in trust leading to increased dependence behavior. However, the trust versus performance results of Rittenberg et al. (2024) indirectly call this into question.

### Purpose

The main purpose of this study was to investigate how trust in automation changes with increasing and decreasing automation reliability and how these changes influence the association between users’ trust and their dependence behavior. This study used methods similar to those of Rittenberg et al. (2024), but with a direct measure of participants’ dependence on the system. Based on the results from Rittenberg et al., it was hypothesized that there would be a gradual decrease in trust in the decreasing reliability group, but no increase in trust in the increasing reliability group but rather a decrease as well. This is because the increasing reliability group will initially interact with automation at a low reliability level, which may hinder the development of trust in the system. If dependence is closely related to trust, we would hypothesize that dependence on the automation would decrease over time in both groups. However, if the performance results of Rittenberg et al. (2024) reflect dependence, dependence may follow reliability level (i.e., more dependence on the automation with higher reliability levels).

## Method

### Participants

One hundred thirty-nine participants were recruited through Amazon Mechanical Turk (MTurk). Participants were required to have normal or corrected-to-normal vision with the ability to discriminate between the colors blue and orange using the Ishihara test (Clark, 1924). Participants were paid $4 CAD for completing the experiment and could receive a $2 CAD performance bonus if they had achieved a performance of over 70% accuracy across all trials.

### Design and Procedures

**Figure 1.**
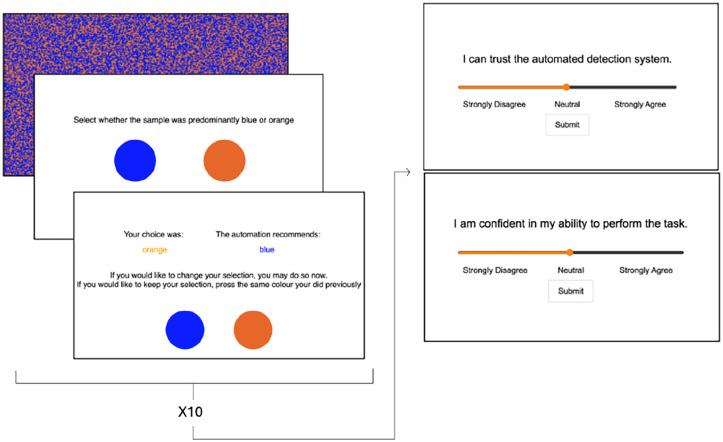
Progression of experimental trial. Slide 1: stimulus. Slide 2: initial color selection. Slide 3: Automation recommendation with option to change initial selection (original selection is also provided). Slide 4 and 5 (right side of figure) are the Trust and Self-Confidence questions, presented every 10 trials.

Participants accessed the experiment through MTurk, a crowdsourcing marketplace run by Amazon. A link redirected participants to the experimental site. The experiment itself was developed using lab.js. Participants began by completing a demographic questionnaire, a color vision test, reading the task instructions and providing informed consent. Participants were told they would be a geological surveyor in a “soil identification task.” This task involved examining six different soil sites to identify acidic and less acidic soil displayed through blue and orange squares. They were told that there was an automated system to help with the task, but it was not always accurate, so they had to survey each sample as well. The images of blue and orange squares were displayed on a 51:49 ratio (Figure 1), similar to the design used by Bartlett and McCarley (2019). Participants were prompted to select whether they believed the image was more blue or more orange by selecting either the blue or orange circle. After giving their initial answer, the automated system displayed its recommendation, to which the participants were able to choose to either agree or disagree with the automated system by providing a final answer.

Participants first completed a practice block of 25 trials without automation, where they were shown images of blue and orange squares and asked to determine if the images were predominantly blue or orange. Following the practice block, participants completed six experimental blocks of 50 trials each, where the reliability of the automated system varied by block. Participants were randomly assigned to one of two groups for these experimental blocks. One group experienced increasing automation reliability and the other had decreasing automation reliability. For the increasing reliability group, the automated system’s reliability started at 50% and increased by 10% increments for each block (i.e., when participants moved to a new soil identification site) until reaching 100% (i.e., 50%, 60%, 70%, 80%, 90%, 100%). Conversely, for the decreasing reliability group, the system’s reliability began at 100% and decreased by 10% increments for each block until it reached 50%.

Participants’ trust in the automated system and their self-confidence were measured through questionnaires, once after the practice block and then five times per experimental block (every ten trials) for a total of 31 times. Both trust and self-confidence were rated on visual analog scales ranging from strongly disagree to strongly agree, where participants used a sliding scale to rate their level of agreeance with the following statements “I can trust the automated detection system” and “I am confident in my abilities to perform this task” (Figure 1).

Participants’ dependence behavior was measured as a proportion of times participants changed their final response from their initial response to agree with the automation’s recommendation by the number of times they had the opportunity to switch their response to agree with the automation (i.e., the number of times their initial response differed from the automation’s recommendation). This measurement was solely focused on participants’ behavior in response to the automation, regardless of whether their responses were correct or incorrect.

Participants’ performance in each block was measured by dividing the number of correct responses for each trial of the color identification task by the total number of trials in the block. This performance measurement was done separately for both initial responses and final responses to evaluate the impact of receiving the automation’s recommendation had on the participants’ overall performance throughout the experiment.

### Data Analysis

Data sets where the overall performance for initial responses was less than 56.7% were excluded because it was likely that these participants were not engaged in the task. After removing these participants, each condition had 40 participants for an overall sample size of 80 participants. Data was formatted using MATLAB (R2022a) and analyzed with R (version 4.1.2). Participants’ trust, self-confidence, dependence behavior, and performance (initial response and final response) were analyzed using a 2 Group (increasing reliability vs. decreasing reliability) × 6 Block mixed ANOVA with a Bonferroni correction (i.e., .05 / 5 = .01 alpha for significance). For instances where sphericity was found to be violated, values were corrected using Greenhouse-Geisser estimates.

## Results

### Dependence

**Figure 2.**
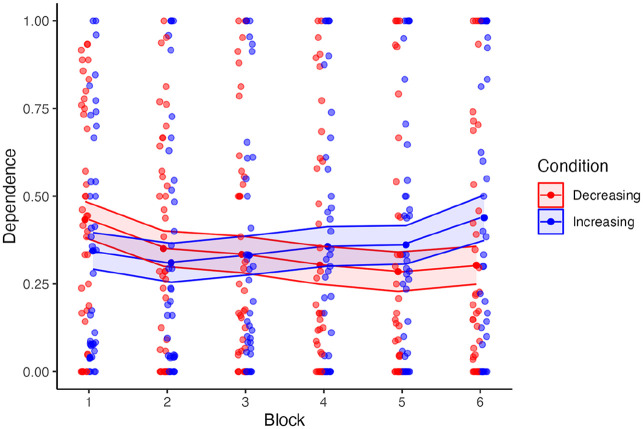
Dependence behavior by block. Dependence was scored on a 0 to 1 scale as the proportion of times participants deviated from their initial response to match the automation’s recommendation. In all results figures, individual points represent a participant’s mean for a given block. The upper and lower limits of the ribbon represent 1 standard error above and below the mean for a given block and condition.

There was no main effect of reliability group on dependence (*F* [1, 78] = 0.10, *p* = .78, *η*^2^*p* = .01), nor a main effect of block on dependence (*F* [3.15, 245.4] = 2.68, *p* = .05, *η*^2^*p* = .03; Figure 2). However, there was a significant interaction effect between reliability group and block on dependence (*F* [3.15, 245.4] = 6.21, *p* < .001, *η*^2^*p* = .07).

A post hoc simple slopes analysis was conducted to examine the relationship between the two variables under both conditions. In the increasing condition, the slope was estimated at 0.02 (*SE* = 0.01, *t* = 1.43, *p* = .15). For the decreasing condition, the slope was estimated at −0.03 (*SE* = 0.01, *t* = −1.93, *p* = .05). While neither slope on its own was significant the interaction emerged due to the different direction of change across blocks, between groups.

### Trust

**Figure 3.**
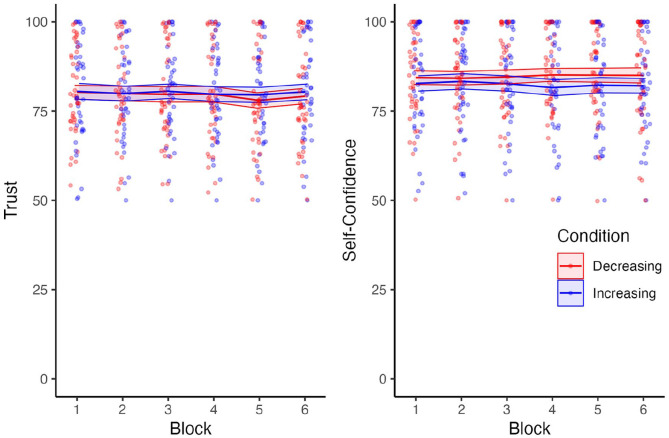
Self-reported trust in the automation and self-confidence across blocks. Both trust and self-confidence were scored on a scale of 0 to 100.

There was no main effect of reliability group on trust (*F* [1, 78] = 0.05, *p* = .82, *η*^2^*p* < .001), nor a main effect of block on trust (*F* [3.59, 280.3] = 1.27, *p* = .29, *η*^2^*p* = .02; Figure 3, Left). There was no interaction between reliability group and block (*F* [3.59, 280.9] = 0.47, *p* = .74, *η*^2^*p* = .006).

### Self-Confidence

There was no significant main effect of reliability group on self-confidence (*F* [1, 78] = 0.69, *p* = .41, *η*^2^*p* = .01; Figure 3, Right). There was no main effect of block on self-confidence (*F* [3.65, 285.1] = 0.07, *p* = .99, *η*^2^*p* < .01). There was no interaction between reliability group and block (*F* [3.65, 285.1] = 0.98, *p* = .41, *η*^2^*p* = .01).

### Performance—Initial Response and Final Response

**Figure 4.**
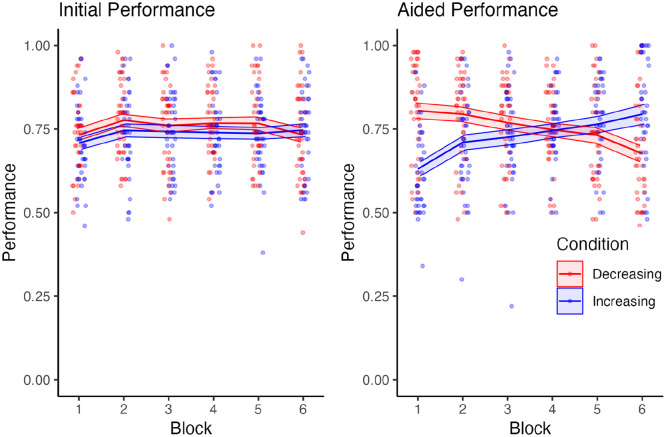
Performance for participants’ initial and final responses (given after automation recommendation) across blocks. Performance was scored on a 0 to 1 scale as the proportion of correct responses in a block.

There was no significant main effect of reliability group on participants’ *initial* response performance (*F* [1, 78] = 0.79, *p* = .38, *η*^2^*p* = .01), however, the effect of group on first response performance approached our Bonferroni corrected alpha (*F* [4.73, 369.0] = 3.12, *p* = .01, *η*^2^*p* = .04) with a trend of the decreasing group having slightly greater performance than the increasing group (Figure 4, Left). There was no interaction between reliability group and block (*F* [4.73, 369.0] = 1.07, *p* = .38, *η*^2^*p* = .01).

There was no significant main effect of reliability group on participants’ *final* response performance (*F* [1, 78] = 0.85, *p* = .36, *η*^2^*p* = .01; Figure 4, Right). There was no main effect of block on participants final response performance (*F* [2.16, 168.1] = 1.88, *p* = .15, *η*^2^*p* = .02). However, there was an interaction between reliability group and block (*F* [2.16, 168.1] = 29.0, *p* < .001, *η*^2^*p* = .27).

## Discussion

This study investigated how trust in automation changes with increasing and decreasing automation reliability and how these changes influence the association between users’ trust and their dependence behavior. Unlike Rittenberg et al. (2024), there was no change in trust with changing reliability level in either group. However, both dependence and final response performance (i.e., after the participants had received advice from the automation) tracked automation reliability level, with dependence and performance increasing over the experiment in the group whose automation increased in reliability and decreased in the group whose automation decreased in reliability.

The lack of a main effect of reliability group on trust suggests that changes in system reliability alone do not always directly impact users’ trust levels. The main difference between the present study and Rittenberg et al. (2024) is that participants provided two responses with the automation’s recommendation given between the two responses to directly measure dependence. This manipulation has been used in previous studies (i.e., where automation provides advice before a user’s decision) to measure dependence and was not expected to influence trust. However, it may be that having the participants provide a response prior to receiving the automation’s advice impacts their perception of the advice in relation to their own performance. Future work should consider when advice from automation is provided.

Rittenberg et al., also showed through mixed effects modeling that participants’ self-confidence impacted the relationship between trust and reliability. While this modeling is beyond the scope of this proceeding, future analyses using mixed effect models are expected to draw out more complex interactions, where an individual’s level of trust and/or self-confidence may impact their dependence behavior.

Despite trust remaining steady, participants’ dependence behavior did change throughout the experiment, with an increasing trend of dependence behavior for the increasing reliability group and a decrease in dependence behavior for the decreasing reliability group. These findings also align with performance for the final responses that changed to match the system’s reliability. Although Rittenberg et al. (2024) did not directly measure dependence, it was suggested that participants may have relied on the automation in at least some trials as their performance in the task tracked the automation’s reliability, which is similar to the results found here. The increase in performance following an increase in reliability may imply that participants depended on the system despite not gaining or losing trust in it. These findings help highlight how dependence on a system can change independently of trust.

The findings showed that self-confidence in performing the task remained relatively stable regardless of changes in automation reliability. This stability in self-confidence might indicate that participants relied on their own abilities consistently, irrespective of the system’s performance. Taken in combination with the fact that most users are not entirely confidence (i.e., the group average being about 0.80 out of 1.00), this could suggest that users lack the required feedback to determine how they were performing. We suspect that providing users with immediate or even delayed feedback could help users calibrate their own abilities with respect to their performance, which would likely influence their trust and dependence on the automated system.

The difference in initial and final response performance suggests that dependence on the automation at low reliability levels is negatively influencing a user’s performance, with the opposite being true of high reliability levels. Although this isn’t a surprising result, it does further support the idea that participants are not capable of assessing one’s own performance, and thus are relying on the automation regardless of whether they had initially responded correctly or incorrectly to the trial.

Several limitations of this study should be noted. The use of MTurk participants may limit the generalizability of the findings to broader populations. Additionally, the study’s task context (color identification) may not fully capture the complexity of real-world automated systems. Future research should explore trust dynamics in more diverse and ecologically valid settings, as well as investigate additional factors influencing trust and dependence, such as user experience and personality traits.

This study highlights the complex relationship between trust, dependence, and automation reliability. Contrary to the findings of Rittenberg et al. (2024), this study found no significant change in trust levels with varying reliability in either group. These results demonstrate that changes in one’s trust are not solely based on system reliability. A main focus of this study was to examine user dependence behavior and its interaction with their trust in the system. It is often thought that trust influences dependence behavior, however this study helps highlight that this is not always the case. Although dependence is often influenced by trust, other factors such as system reliability and the user’s self-confidence also play a role in decision-making.
